# 2015/16 I‐MOVE/I‐MOVE+ multicentre case‐control study in Europe: Moderate vaccine effectiveness estimates against influenza A(H1N1)pdm09 and low estimates against lineage‐mismatched influenza B among children

**DOI:** 10.1111/irv.12520

**Published:** 2018-03-14

**Authors:** Esther Kissling, Marta Valenciano, Francisco Pozo, Ana‐Maria Vilcu, Annicka Reuss, Caterina Rizzo, Amparo Larrauri, Judit Krisztina Horváth, Mia Brytting, Lisa Domegan, Monika Korczyńska, Adam Meijer, Ausenda Machado, Alina Ivanciuc, Vesna Višekruna Vučina, Sylvie van der Werf, Brunhilde Schweiger, Antonino Bella, Alin Gherasim, Annamária Ferenczi, Katherina Zakikhany, Joan O′Donnell, Iwona Paradowska‐Stankiewicz, Frederika Dijkstra, Raquel Guiomar, Mihaela Lazar, Sanja Kurečić Filipović, Kari Johansen, Alain Moren, I‐MOVE/I‐MOVE+ study team

**Affiliations:** ^1^ EpiConcept Paris France; ^2^ National Centre for Microbiology Instituto de Salud Carlos III Madrid Spain; ^3^ Sorbonne Universités UPMC Univ Paris 06 INSERM Institut Pierre Louis d’épidémiologie et de Santé Publique (IPLESP UMRS 1136) Paris France; ^4^ Department for Infectious Disease Epidemiology Robert Koch Institute Berlin Germany; ^5^ Department of Infectious Disease Istituto Superiore di Sanità Rome Italy; ^6^ National Centre for Epidemiology Instituto de Salud Carlos III Madrid Spain; ^7^ Ciber Epidemiología y Salud Pública (CIBERESP) Madrid Spain; ^8^ National Centre for Epidemiology Budapest Hungary; ^9^ The Public Health Agency of Sweden Stockholm Sweden; ^10^ Health Service Executive ‐ Health Protection Surveillance Centre Dublin Ireland; ^11^ National Institute of Public Health‐National Institute of Hygiene Warsaw Poland; ^12^ Centre for Infectious Disease Control National Institute for Public Health and the Environment Bilthoven The Netherlands; ^13^ Instituto Nacional de Saúde Dr Ricardo Jorge Lisbon Portugal; ^14^ Development for Microbiology and Immunology Cantacuzino Institute National Institute of Research Bucharest Romania; ^15^ Croatian Institute of Public Health Zagreb Croatia; ^16^ Institut Pasteur Paris France; ^17^ National Reference Centre for Influenza Robert Koch Institute Berlin Germany; ^18^ European Centre for Disease Prevention and Control (ECDC) Stockholm Sweden

**Keywords:** case‐control study, influenza, influenza vaccine, multicentre study, vaccine effectiveness

## Abstract

**Background:**

During the 2015/16 influenza season in Europe, the cocirculating influenza viruses were A(H1N1)pdm09 and B/Victoria, which was antigenically distinct from the B/Yamagata component in the trivalent influenza vaccine.

**Methods:**

We used the test‐negative design in a multicentre case‐control study in twelve European countries to measure 2015/16 influenza vaccine effectiveness (VE) against medically attended influenza‐like illness (ILI) laboratory‐confirmed as influenza. General practitioners swabbed a systematic sample of consulting ILI patients and a random sample of influenza‐positive swabs was sequenced. We calculated adjusted VE against influenza A(H1N1)pdm09, A(H1N1)pdm09 genetic group 6B.1 and influenza B overall and by age group.

**Results:**

We included 11 430 ILI patients, of which 2272 were influenza A(H1N1)pdm09 and 2901 were influenza B cases. Overall VE against influenza A(H1N1)pdm09 was 32.9% (95% CI: 15.5‐46.7). Among those aged 0‐14, 15‐64 and ≥65 years, VE against A(H1N1)pdm09 was 31.9% (95% CI:** −**32.3 to 65.0), 41.4% (95% CI: 20.5‐56.7) and 13.2% (95% CI:** −**38.0 to 45.3), respectively. Overall VE against influenza A(H1N1)pdm09 genetic group 6B.1 was 32.8% (95% CI:** −**4.1 to 56.7). Among those aged 0‐14, 15‐64 and ≥65 years, VE against influenza B was **−**47.6% (95% CI:** −**124.9 to 3.1), 27.3% (95% CI:** −**4.6 to 49.4) and 9.3% (95% CI:** −**44.1 to 42.9), respectively.

**Conclusions:**

Vaccine effectiveness (VE) against influenza A(H1N1)pdm09 and its genetic group 6B.1 was moderate in children and adults, and low among individuals ≥65 years. Vaccine effectiveness (VE) against influenza B was low and heterogeneous among age groups. More information on effects of previous vaccination and previous infection is needed to understand the VE results against influenza B in the context of a mismatched vaccine.

## 1 INTRODUCTION

In February 2015, WHO recommended that the 2015/16 Northern Hemisphere trivalent influenza vaccine should include the same influenza A(H1N1)pdm09 strain as the 2014/15 season vaccine (the same component for the trivalent vaccine since the 2010/11 season), but different influenza A(H3N2) and B components, namely a virus from the 3C.3a A(H3N2) genetic group and the genetic group 3 of the B/Yamagata lineage. The recommended strains were as follows: an influenza A/California/7/2009 (H1N1)pdm09‐like virus, an influenza A/Switzerland/9715293/2013 (H3N2)‐like virus and an influenza B/Phuket/3073/2013‐like Yamagata lineage virus.

An interim analysis for the 2015/16 season published in early February 2016 from the European I‐MOVE/I‐MOVE+ multicentre case‐control study showed a predominance of A(H1N1)pdm09 (71%, 246/348), with influenza B cocirculating (22%; 77/348) among participating study sites.^1^ Among the B specimens where lineage information was available, 97.3% (36/37) were of the B/Victoria lineage, indicating a mismatch with the influenza B/Yamagata virus included in the trivalent vaccine.

In this eighth season of the I‐MOVE/I‐MOVE+ multicentre case‐control study, we aimed to measure end‐of‐season 2015/16 vaccine effectiveness against influenza A(H1N1)pdm09 and influenza B, by age group, vaccine type, by prior (2014/15) vaccination status and by time since vaccination and for the total population and the target group for vaccination.

Nine of twelve study sites also participated in a pilot laboratory project where they randomly selected specimens for sequencing of at least the gene segment coding for the haemagglutinin, in order to compute a representative VE estimate against the influenza A(H1N1)pdm09 6B.1 genetic group.

## 2 METHODS

Twelve European study sites located in Croatia, France, Germany, Hungary, Ireland, Italy, Poland, Portugal, Romania, Spain, Sweden and the Netherlands participated in the test‐negative 2015/16 multicentre case‐control study. The methods have been described previously^2^, ^3^, ^4^ and are based on the ECDC generic case‐control study protocol and the I‐MOVE+ protocol.^5^, ^6^

Participating practitioners interviewed and collected nasopharyngeal or combined naso‐ and oropharyngeal specimens from a systematic sample of consenting patients seeking medical attention for influenza‐like illness (ILI). In Hungary, only patients aged 18 years and older and in Croatia only patients aged 65 years and older were eligible. Practitioners collected in a standardised report form information including symptoms, date of onset and swabbing, 2015/16 seasonal vaccination status, date of influenza vaccination and vaccine product, prior (2014/15) seasonal vaccination status, sex, age and presence of chronic medical conditions in the past 12 months.

Seven study sites included a question on belonging to the target group for vaccination. In France, Germany, Poland, Portugal and Sweden, the target group was defined from patients’ information on age, chronic conditions and pregnancy. Additionally, in Portugal, being a health professional or carer and a cohabitant or carer of a patient at risk aged less than 6 months and in Poland, belonging to an occupational risk group (eg, healthcare worker), defined the target group.

In the pooled analysis, we included patients meeting the European Union ILI case definition,^7^ swabbed within 7 days of symptom onset, and who had not received antivirals in the 14 days prior to swabbing.

A case of confirmed influenza was an ILI patient who was swabbed and tested positive for influenza virus using real‐time reverse‐transcription polymerase chain reaction (RT‐PCR). Controls were ILI patients who tested negative for any influenza virus using RT‐PCR.

We defined a person as vaccinated if he or she had received at least one dose of a 2015/16 seasonal influenza vaccine more than 14 days before ILI symptom onset. Those vaccinated less than 15 days before ILI onset were excluded. All other patients were classified as unvaccinated.

For each study site, we included ILI patients presenting more than 14 days after the start of national or regional influenza vaccination campaigns and we excluded controls presenting before the onset week of the first influenza type/subtype‐specific case. ILI patients presenting in weeks of onset after two or more consecutive weeks of no cases and influenza A cases that were not further subtyped were also excluded from the analysis.

For each study site, we computed the odds ratio (OR) of being vaccinated in cases vs controls. We conducted a complete analysis excluding patients with missing values for any of the variables in the model measuring adjusted VE. Using Cochran's Q‐test and the *I* ^2^ index, we tested the heterogeneity between study sites.^8^ We estimated the pooled type/subtype influenza VE as (1 minus the OR)*100 using a one‐stage model with study site as a fixed effect.

Using a logistic regression model, we calculated VE including potential confounding factors: date of symptom onset (modelled as a restricted cubic spline with 4 knots where sample size allowed), age (modelled as a restricted cubic spline with 4 knots or age groups depending on the analysis), sex and presence of at least one chronic medical condition (including pregnancy and obesity where available). We used the one in ten rule of predictor degrees of freedom to events to determine the maximum number of covariates to include in analyses with low sample sizes in order to avoid overfitting the model.^9^, ^10^

To study the effect of prior (2014/15) vaccination on the 2015/16 VE, we conducted an indicator analysis using four categories: individuals unvaccinated in both seasons (reference category), vaccinated in 2014/15 only, vaccinated in 2015/16 only and vaccinated in both seasons. We did not measure effect of prior (2014/15) vaccination among children aged <9 years as their vaccination definition is based on previous vaccination history (children older than 6 months and less than 9 years old who have not been vaccinated in the previous influenza season should receive two doses of the seasonal influenza vaccine). We also conducted a stratified analysis, measuring VE of the 2015/16 vaccine among those vaccinated in 2014/15 and separately among those not vaccinated in 2014/15.

We measured VE by age group (0‐14, 15‐64 and 65 years and older), by type of vaccine (inactivated subunit and inactivated split virion) and in the target group for vaccination. We tested for interaction between vaccination and age group, chronic medical condition, onset month and sex, using the likelihood ratio test to compare the additive model with the interaction.

To study the effects of waning on the vaccine effect within a season, we further estimated VE by time since vaccination, modelling days between vaccination and symptom onset dates as a restricted cubic spline with 4 knots.^11^ In this analysis, we additionally included patients vaccinated 14 days or less before symptom onset (excluded from the main analysis).

Nine study sites participated in a laboratory pilot project (DE, FR, HU, IE, PT, RO, SE, ES and NL) for sequencing at least the haemagglutinin gene segment for each influenza type/subtype. In this laboratory pilot project, either all specimens were selected for sequencing or a proportion of specimens were randomly selected for sequencing to ensure representativity. The proportion of specimens randomly selected for sequencing could vary over time (eg, higher early in the season and lower during the peak) and a sampling fraction was calculated for each study site and time unit. The specimens were sent to the corresponding National Influenza Centre, where influenza diagnosis was confirmed, and viruses were characterised by sequencing the HA1 coding portion of the haemagglutinin gene. Analysis of the nucleotide and amino acid sequences of the HA1 coding portion of the haemagglutinin gene was performed in MEGA6 to determine clade distribution.

We weighted the genetic group‐specific VE analysis using the reciprocal of the sequencing sampling fraction for each time period and study site and used robust standard errors.

Data management and statistical analyses were carried out using Stata 14 (StataCorp. 2015. College Station, TX, USA).

## 3 RESULTS

**Figure 1:**
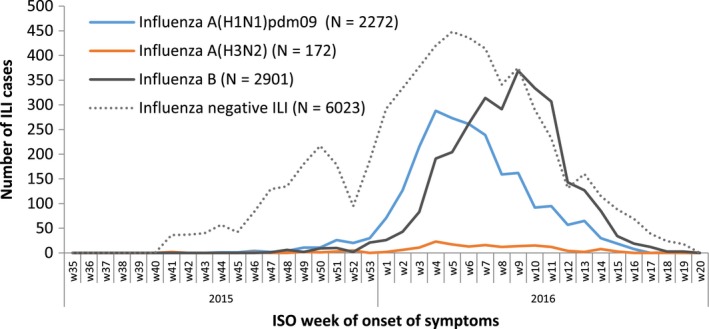
Number of influenza‐like illness (ILI) reports by case status, week of symptom onset and influenza virus type/subtype, total population, I‐MOVE/I‐MOVE+ multicentre case‐control study, influenza season 2015/16, weeks 35/2015‐week 20/2016 (study period with influenza‐positive cases: week 41/2015‐week 19/2016)

The 2015/16 influenza season in Europe was characterised by the cocirculation of influenza A(H1N1)pdm09 and influenza B viruses (Figure 1). Influenza A(H3N2) viruses circulated at very low levels. The study period ranged from week 44/2015 to week 18/2016 for influenza A(H1N1)pdm09 with cases peaking in week 4/2016 and from week 45/2015 to week 19/2016 for influenza B, with cases peaking in week 9/2016.

**Figure 2:**
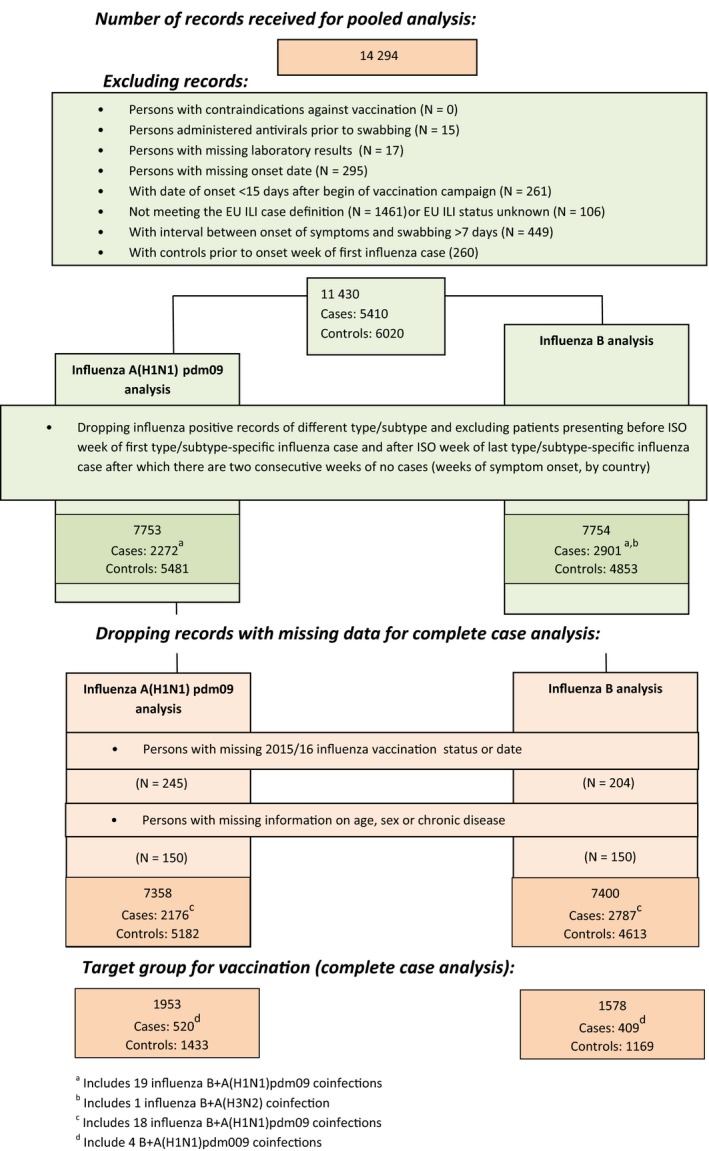
Flow chart of data exclusion for pooled analysis. I‐MOVE/I‐MOVE+ multicentre case‐control study, influenza season 2015/16 (week 41/2015‐week 19/2016)

Of the 14 294 ILI patients recruited, 11 430 met the eligibility criteria (5410 cases and 6020 controls). In the influenza type/subtype‐specific analysis, 2272 cases of influenza A(H1N1)pdm09 and 2901 cases of influenza B were included (Figure 2). We did not include the 172 patients testing positive for influenza A(H3N2) in the analysis due to small sample size.

The proportion vaccinated with the 2015/16 influenza vaccine was 9.7% among controls, 6.7% among influenza A(H1N1)pdm09 cases and 6.3% among influenza B cases (Table 1).

The median age of influenza A(H1N1)pdm09 cases was 35 years, of controls 29 years and of influenza B cases 12 years (Table 2). Compared to influenza A(H1N1)pdm09, a higher proportion of influenza B cases were less than 15 years (55.3% vs 30.3%) and a lower proportion were 15‐64 years old (40.8% vs 63.5%). The proportion of patients aged 65 and older varied between controls, influenza A(H1N1)pdm09 and influenza B cases with 9.5%, 6.2% and 3.9%, respectively.

**Table 1:** Details for influenza A(H1N1)pdm09 (n = 2272) and influenza B cases (n = 2901) and controls (n = 1650) included in the 2015/16 season influenza vaccine effectiveness analysis (week 41/2015‐week 19/2016), I‐MOVE/I‐MOVE+ multicentre case‐control study

Variables	Number of test‐negative controls a/total n (%)	Number of influenza A(H1N1)pdm09/total n (%)	Number of influenza B cases/total n (%)
Median age (years)	29.0	35.0	12.0
Age groups			
0‐4	1437/6004 (23.9)	365/2268 (16.1)	536/2894 (18.5)
5‐14	739/6004 (12.3)	321/2268 (14.2)	1064/2894 (36.8)
15‐64	3255/6004 (54.2)	1441/2268 (63.5)	1182/2894 (40.8)
≥65	573/6004 (9.5)	141/2268 (6.2)	112/2894 (3.9)
Missing	16	4	7
Sex			
Female	3159/5975 (52.9)	1137/2259 (50.3)	1456/2871 (50.7)
Missing	45	13	30
Days between onset of symptoms and swabbing			
0	389/6020 (6.5)	95/2272 (4.2)	126/2901 (4.3)
1	2008/6020 (33.4)	824/2272 (36.3)	907/2901 (31.3)
2	1589/6020 (26.4)	663/2272 (29.2)	899/2901 (31.0)
3	934/6020 (15.5)	348/2272 (15.3)	539/2901 (18.6)
4‐7	1100/6020 (18.3)	342/2272 (15.1)	430/2901 (14.8)
Seasonal vaccination, 2015/16	564/5802 (9.7)	150/2223 (6.7)	180/2841 (6.3)
Vaccinated <15 d before onset of symptoms	17	0	0
Missing	201	49	60
Prior season influenza vaccination^b^			
Not vaccinated in any season	3259/3896 (83.6)	1421/1593 (89.2)	1481/1635 (90.1)
Current season (2015/16) vaccination only	87/3896 (2.2)	17/1593 (1.1)	19/1635 (1.2)
Prior (2014/15) season vaccination only	138/3896 (3.5)	39/1593 (2.4)	33/1635 (2.0)
Current and prior season vaccination	412/3896 (10.6)	116/1593 (7.3)	102/1635 (6.2)
Missing or vaccinated <15 d before onset	279	109	69
Seasonal vaccination type			
Not vaccinated	5255/5819 (90.3)	2073/2203 (93.3)	2661/2809 (93.7)
Inactivated subunit	204/5819 (3.5)	57/2203 (2.6)	68/2809 (2.4)
Inactivated split virion trivalent	202/5819 (3.5)	64/2203 (2.9)	71/2809 (2.5)
Adjuvanted^c^	60/5819 (1.0)	6/2203 (0.3)	6/2809 (0.2)
Inactivated cell‐derived subunit	1/5819 (0.0)	0/2203 (0.0)	0/2809 (0)
Quadrivalent vaccine^d^	3/5819 (0.1)	3/2203 (0.1)	3/2809 (0.1)
Unknown vaccine type	94/5819 (1.6)	20/2203 (0.9)	32/2809 (1.1)
Missing vaccination status or date or vaccinated <15 d before onset	81	49	60
At least one chronic condition	1194/5900 (20.2)	391/2227 (17.6)	341/2870 (11.9)
Missing	120	45	31
At least one hospitalisation in the previous 12mo for chronic conditions	110/5857 (1.9)	26/2214 (1.2)	21/2854 (0.7)
Missing	163	58	47
Belongs to the target group for vaccination	1648/5931 (27.8)	544/2236 (24.3)	434/2873 (15.1)
Missing	89	36	28
Study sites			
Croatia	39/6020 (0.6)	15/2272 (0.7)	19/2901 (0.7)
France	1471/6020 (24.4)	508/2272 (22.4)	1294/2901 (44.6)
Germany	1726/6020 (28.7)	436/2272 (19.2)	571/2901 (19.7)
Hungary	593/6020 (9.9)	54/2272 (2.4)	112/2901 (3.9)
Ireland	241/6020 (4.0)	181/2272 (8)	130/2901 (4.5)
Italy	498/6020 (8.3)	34/2272 (1.5)	390/2901 (13.4)
Poland	312/6020 (5.2)	136/2272 (6.0)	65/2901 (2.2)
Portugal	186/6020 (3.1)	111/2272 (4.9)	11/2901 (0.4)
Romania	80/6020 (1.3)	61/2272 (2.7)	0/2901 (0.0)
Spain	286/6020 (4.8)	447/2272 (19.7)	165/2901 (5.7)
Sweden	376/6020 (6.2)	175/2272 (7.7)	65/2901 (2.2)
The Netherlands	212/6020 (3.5)	114/2272 (5.0)	79/2901 (2.7)

a Controls for “any influenza” used here (number of controls differs slightly for influenza A(H1N1)pdm09 and B analyses, due to the inclusion criteria).

b Among patients aged 9 y and over.

c Includes squalene (MF59), virosome and aluminium phosphate gel adjuvants.

d Includes Fluenz Tetra (nasal spray) as well as Fluarix Tetra (injectable).

The proportion of patients with at least one chronic condition was similar between controls and influenza A(H1N1)pdm09 cases (20.2% and 17.6%, respectively), but lower among influenza B cases (11.9%).

Among controls, 81.7% were swabbed within 3 days of symptom onset compared to 84.9% and 85.2% of influenza A(H1N1)pdm09 and influenza B cases, respectively. Among controls, 6.5% were swabbed on the day of symptom onset, compared to 4.2% and 4.3% of influenza A(H1N1)pdm09 and influenza B cases, respectively.

In total, 10.6% of controls had received both the 2014/15 and the 2015/16 vaccines compared to 7.3% and 6.2% of influenza A(H1N1)pdm09 and B cases, respectively. The proportion of unvaccinated in the current and previous season was 89.2% for influenza A(H1N1)pdm09 cases, 90.1% for influenza B cases and 83.6% for controls.

Information on vaccine type received was available for 470 (83.3%) of vaccinated controls, 130 (86.7%) vaccinated influenza A(H1N1)pdm09 and 149 (82.2%) vaccinated influenza B cases. Trivalent inactivated subunit and trivalent inactivated split virion vaccines were used among 43.4% and 43.0% of vaccinated controls, 43.8% and 49.2% of vaccinated influenza A(H1N1)pdm09 cases and 45.9% and 48.0% of vaccinated influenza B cases, respectively.

From the 11 430 patients meeting the eligibility criteria, we further excluded patients with missing information on 2015/16 seasonal vaccination status or date, onset/swab date, age, sex or presence of chronic condition. We included 7358 patients for the complete case analysis of VE against influenza A(H1N1)pdm09 and 7400 patients for the analysis against influenza B among all ages (Figure 2). For the complete case analysis restricted to the target group for vaccination, we included 1953 patients (520 influenza A(H1N1)pdm09 cases) in the analysis of VE against A(H1N1)pdm09 and 1578 patients (409 influenza B cases) in the analysis of VE against influenza B.

### 3.1 Influenza A(H1N1)pdm09

Statistical heterogeneity between VE estimates against influenza A(H1N1)pdm09 by study site was low overall (among all ages) and among those aged 15‐64 years (*I* ^2^ index 0% and 10%, respectively). Due to small sample sizes, it was not possible to estimate heterogeneity among other age groups.

The adjusted VE in the total population (all ages) against influenza A(H1N1)pdm09 was 32.9% (95% CI: 15.5‐46.7) (Table 2). The adjusted VE against influenza A(H1N1)pdm09 was 31.9% (95% CI: **−**32.3 to 65.0) among the 0‐ to 14‐year‐olds and 41.4% (95% CI: 20.5‐56.7) among the 15‐ to 64‐year‐olds. Among the target group for vaccination, VE (all ages) was 33.0% (95% CI: 10.8‐49.7). It was 55.5% (95% CI: **−**35.1 to 85.3) and 42.9% (95% CI: 14.5‐61.9) among those aged 0‐14 and 15‐64 years, respectively. Among those aged 65 years and older, VE adjusted for age and study site was 13.2% (95% CI: **−**38.0 to 45.3).

**Table 2:** Pooled crude and adjusted seasonal vaccine effectiveness against laboratory‐confirmed influenza by influenza type/subtype and A(H1N1)pdm09 genetic group 6B.1, overall, by age groups, by previous vaccination status and for the target group for vaccination. I‐MOVE/I‐MOVE+ multicentre case‐control study, influenza season 2015/16 (week 41/2015‐week 19/2016)

Type/subtype	Analysis scenario			N^a^	Cases; vacc/Controls; vacc^a^	Crude VE^a^^,^ b	CI	Adjusted VE^c^	CI
A(H1N1)pdm09^d^	By age	All ages		7358	2176;148/5182;527	41.9	28.9‐52.6	32.9	15.5‐46.7
		0‐14 y		2424	648;14/1776;56	25.4	**−**39.1 to 60.0	31.9	**−**32.3 to 65.0
		15‐64 y		4308	1394;73/2914;230	40.8	21.1‐55.6	41.4	20.5‐56.7
		65+ y		625	134;61/491;240	26.8	**−**14.4 to 53.1	13.2^e^	**−**38.0 to 45.3
	Target group for vaccination	All ages		1953	520;114/1433;425	44.3	27.4‐57.2	33.0	10.8‐49.7
		0‐14 y		253	70;6/183;24	48.7	**−**46.7 to 82.1	55.5^f^	**−**35.1 to 85.3
		15‐64 y		1061	315;47/746;155	45.2	18.9‐62.9	42.9	14.5‐61.9
	By vaccine type—all ages	Unvaccinated (ref)		6683	2028/4655				
		Subunit vaccine		242	57/185	39.3	16.2‐56.1	33.9	6.7‐53.1
		Split virion vaccine		255	62/193	47.6	28.6‐61.5	36.3	10.8‐54.5
	By vaccine type—0‐ to 14‐y‐olds	Unvaccinated (ref)		2354	634/1720	Ref			
		Subunit vaccine		24	4/20	46.2	**−**68.5 to 82.8	51.1	**−**55.8 to 84.6
		Split virion vaccine		28	6/22	7.6	**−**144.8 to 65.1	16.3	**−**137.2 to 70.4
	By vaccine type—15‐ to 64‐y‐olds	Unvaccinated (ref)		4005	1321/2684	Ref			
		Subunit vaccine		112	27/85	43.5	9.9‐64.6	45.6	12.1‐66.4
		Split virion vaccine		106	28/78	45.7	14.2‐65.6	45.2	11.8‐65.9
	By prior (2014/15) influenza vaccination status—≥9‐y‐olds	Neither		4378	1404/2974	Ref		Ref	
		2015/16 season only		100	17/83	59.2	28.8‐76.6	54.7	19.6‐74.5
		2014/15 season only		146	38/108	19.0	**−**20.3 to 45.5	8.0	**−**39.3 to 39.2
		Study and previous season		497	114/383	43.0	28.0‐54.9	28.4	6.2‐45.4
	By prior (2014/15) influenza vaccination status—≥9‐y‐olds, target group	Neither		1106	335/771				
		2015/16 season only		67	10/57	66.5	30.8‐83.8	60.4	16.0‐81.3
		2014/15 season only		85	14/71	53.6	12.8‐75.3	46.4	**−**2.4 to 72.0
		Study and previous season		428	95/333	46.4	28.0‐60.1	31.8	5.7‐50.7
	By prior (2014/15) influenza vaccination status—15‐ to 64‐y‐olds	Neither		3707	1244/2483				
		2015/16 season only		66	10/56	70.3	39.5‐85.4	68.2	34.4‐84.6
		2014/15 season only		102	30/72	11.0	**−**40.1 to 43.4	12.7	**−**38.9 to 45.1
		Study and previous season		220	58/162	32.4	6.5‐51.2	32.1	4.2‐51.8
	A(H1N1)pdm09 clade 6B.1	All ages		4779	645;46/4134;434	45.5	18.4‐63.5	32.8	**−**4.1 to 56.7
		0‐14 y		1505	191;5/1314;43	38.6	**−**74.8 to 78.4	51.3	**−**33.5 to 82.3
		15‐64 y		2840	417;19/2423;197	42.5	**−**8.2 to 69.4	40.1	**−**12.9 to 68.3
		65+ y (sample size too small)		406	36;22/370;189				
Influenza B^f^	By age	0‐14 y		3304	1545;82/1759;52	**−**81.4	**−**159.2 to 26.9	**−**47.6	**−**124.9 to 3.1
		15‐64 y		3606	1138;49/2468;196	46.4	25.4‐61.5	27.3	**−**4.6 to 49.4
		65+ y		488	104;46/384;186	9.3	**−**44.1 to 42.9		
	Target group for vaccination	0‐14 y		326	141;34/185;27	**−**21.3	**−**121.0 to 33.4	1.7^g^	**−**94.5 to 50.3
		15‐64 y		751	163;22/588;121	46.8	11.4‐68.1	38.4	**−**6.6 to 64.4
	By vaccine type	0‐ to 14‐y‐olds	Unvaccinated (ref)	3170	1463/1707				
			Subunit vaccine	50	31/19	‐93.7	**−**246.0 to 8.4	**−**56.4	**−**202.1 to 19.0
			Split virion vaccine	67	44/23	**−**106.9	**−**245.0 to 24.1	**−**83.5	**−**232.9 to 1.1
		15‐ to 64‐y‐olds	Unvaccinated (ref)	3361	1089/2272				
			Subunit vaccine	89	17/72	47.1	8.4‐69.4	17.7	**−**48.0 to 54.3
			Split virion vaccine	84	15/69	57.9	25.2‐76.3	44.4	**−**2.8 to 70.0
	By prior (2014/15) influenza vaccination status	15‐ to 64‐y‐olds	Neither	3194	1055/2139				
			2015/16 season only	59	13/46	37.8	**−**17.1 to 67.0	28.3	**−**40.2 to 63.3
			2014/15 season only	77	16/61	49.1	10.0‐71.2	41.3	**−**8.7 to 68.3
			Both seasons	176	35/141	49.0	24.8‐65.4	23.7	**−**16.8 to 50.2

a Based on the complete case analysis, records with missing age, sex, chronic condition, vaccination status are dropped.

b Crude VE adjusted by study site.

c Data adjusted for age (restricted cubic spline or age group), onset date (restricted cubic spline), sex, chronic condition and study site unless otherwise indicated.

d Study sites included in A(H1N1)pdm09 all ages and ≥9 y analysis: DE, ES, FR, HR, HU, IE, IT, NL, PL, PT, RO, SE; HU and HR not included in A(H1N1)pdm09 0‐14 y analysis; HR not included in A(H1N1)pdm09 15‐64 y analysis; study sites included in A(H1N1)pdm09 clade 6B.1 analysis of all ages and 15‐ to 64‐y‐olds: DE, ES, FR, HU, IE, NL, PT, RO, SE; HU not included in A(H1N1)pdm09 clade 6B.1 0‐14 y analysis; study sites included in B 0‐14 y analysis: DE, ES, FR, IE, IT, NL, PL, PT, SE; study sites included in B 15‐64 y analysis: DE, ES, FR, HU, IE, IT, NL, PL, PT, SE; study sites included in B 65 y and older analysis: DE, ES, FR, HR, HU, IE, IT, NL, PL, SE.

e Adjusted by age and study site only.

f Due to heterogeneity of VE estimates against influenza B between age groups, no “all ages” estimate against influenza B was attempted.

g Adjusted by time and study site only.

**Table 3:** Influenza A(H1N1)pdm09, influenza B/Yamagata and influenza B/Victoria viruses characterised by clade and study site, study sites participating in the laboratory pilot study, I‐MOVE multicentre case‐control study, Europe, influenza season 2015/6 (week 41/2015‐week 16/2016)

Characterised viruses	Genetic group	DE	FR	HU	IE	PT	RO	ES	SE	NL	Total (%)
Influenza A(H1N1)pdm09											
A/England/377/2015	6B.2	3	8	0	0	0	2	1	1	0	15 (2.1)
A/SouthAfrica/3626/2013	6B	4	1	1	1	12	2	30	0	5	56 (7.7)
A/Slovenia/2903/2015	6B.1	143	83	19	15	76	27	245	20	24	652 (90.2)
Total		150	92	20	16	88	31	276	21	29	723
Influenza B											
B/Phuket/3073/2013	3	11	0	0	0	‐	‐	0	1	0	12 (3.7)
B/Brisbane/60/2008	1A	135	85	32	5	‐	‐	9	15	28	309 (96.3)
Total		146	85	32	5	‐	‐	9	16	28	321

The adjusted VE for trivalent inactivated subunit vaccine against influenza A(H1N1)pdm09 (all ages) was 33.9% (95% CI: 6.7‐53.1) and for trivalent inactivated split virion vaccine 36.9% (95% CI: 10.8‐54.5) (Table 2).

Information on prior vaccination status was missing among 6.7% of ILI patients (restricting to those 9 years and older). When using the indicator analysis, with the reference of those not vaccinated in the current or previous season, the VE among those aged 9 years and older against influenza A(H1N1)pdm09 was 54.7% for those who received 2015/16 seasonal influenza vaccine only (95% CI: 19.6‐74.5), 8.0 (95% CI: **−**39.3 to 39.2) for those who received prior (2014/15) vaccine only and 28.4% (95% CI: 6.2‐45.4) for those who received both 2015/16 and 2014/15 season vaccine (Table 2).

In the stratified analysis, the VE of current influenza vaccination against A(H1N1)pdm09 among those aged 9 years and older was 56.2% (full model adjusted, 95% CI: 22.2‐75.3) among those not vaccinated in 2014/15 and 6.9% (adjusted by age and study size, 95% CI: **−**51.5 to 42.8) among those vaccinated in 2014/15.

**Figure 3:**
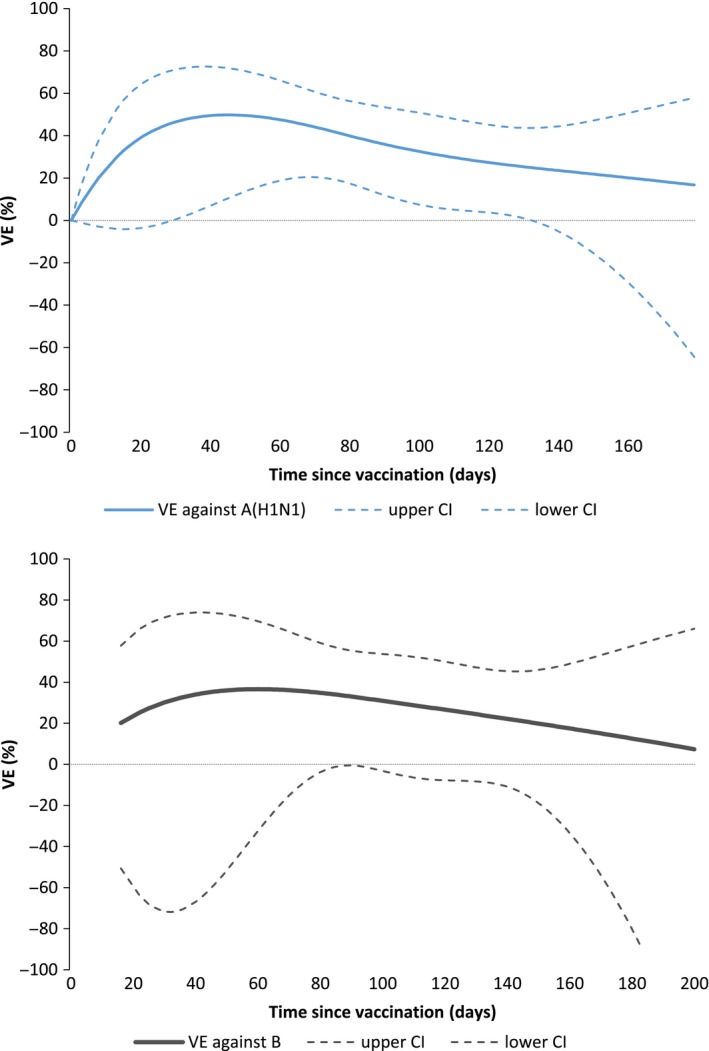
Adjusted vaccine effectiveness (VE) and 95% CI against influenza A(H1N1)pdm09 (all ages) and influenza B (15 y and older) by time since vaccination, total population, I‐MOVE/I‐MOVE+ multicentre case‐control study, influenza season 2015/16 (week 41/2015‐week 19/2016)

When modelling VE by time since vaccination, VE against influenza A(H1N1)pdm09 among all ages increased to 49.8% at 45 days since vaccination and declined to 9.3% at 218 days since vaccination (Figure 3).

During the study period where specimens were sequenced, the nine sites participating in the laboratory pilot season genetically characterised 723 of 2087 (34.6%) influenza A(H1N1)pdm09 specimens among all ages. Of these, 15 (2.1%) belonged to the genetic group represented by A/England/377/2015 (genetic group 6B.2), 56 (7.7%) to the genetic group represented by A/SouthAfrica/3626/2013 (genetic group 6B) and 652 (90.2%) to the genetic group represented by A/Slovenia/2903/2015 (genetic group 6B.1)(Table 3). The adjusted VE against 6B.1 was 32.8% (95% CI: **−**4.1 to 56.7) overall for all age groups, 51.3% (95% CI: **−**33.5 to 82.3) among the 0‐ to 14‐year‐old and 40.1% (95% CI: **−**12.9 to 68.3) among 15‐ to 64‐year‐old age groups (Table 2). The sample size was too small to calculate VE for those aged 65 years and older.

### 3.2 Influenza B

The *I* ^2^ index for heterogeneity between VE estimates against influenza B by study site was 56% among all ages and 0% among those aged 15‐64 years. Due to small sample size, it was not possible to estimate heterogeneity among those aged 65 years and older. Among children, we could measure the *I* ^2^ between three countries (DE, FR and IT; in all other countries, less than 5 children were vaccinated), which was 0%.

The adjusted VE against influenza B was **−**47.6% (95% CI: **−**124.9 to 3.1) among the 0‐ to 14‐year‐olds and 27.3% (95% CI: **−**4.6 to 49.4) among the 15‐ to 64‐year‐olds (Table 2). Crude VE was 9.3% (95% CI: **−**44.1 to 42.9) among those aged 65 years and older (all belong to the target group for vaccination only), and the small sample size did not allow for adjusted VE estimates. The chi‐square of the likelihood ratio test for interaction between vaccine and age group was 24.0 (*P* < .001). Due to this strong interaction between age group and vaccine, we did not attempt to calculate an overall (all ages) VE. The adjusted VE among the target group for vaccination was 1.7% (95% CI: **−**94.5 to 50.3) and 38.4% (95% CI: **−**6.6 to 64.4) among those aged 0‐14 and 15‐64 years, respectively.

The adjusted VE for trivalent inactivated subunit vaccine against influenza B among those aged 0‐14 years was **−**56.4% (95% CI: **−**202.1 to 19.0) and for split virion vaccine **−**83.5% (95% CI: **−**232.9 to 1.1) (Table 3). For those aged 15‐64 years, it was 17.7% (95% CI: **−**48.0 to 54.3) for subunit vaccine and 44.4% (95% CI: **−**2.8 to 70.0) for split virion vaccine.

Information on prior vaccination status was missing in 5.1% of ILI patients (restricting to those 9 years and older). When using the indicator analysis, with the reference of those not vaccinated in the current or previous season, the VE among 15‐ to 64‐year‐olds receiving the current 2015/16 seasonal influenza vaccine only was 28.3% (95% CI: **−**40.2 to 63.3), 41.3 (95% CI: **−**8.7 to 68.3) among those receiving prior season (2014/15) vaccine only and 23.7% (95% CI: **−**16.8 to 50.2) among those who received both 2015/16 and prior season (2014/15) vaccine (Table 2).

In the stratified analysis, the VE of current influenza vaccination against influenza B among 15‐ to 64‐year‐olds was 28.7% (95% CI: **−**39.6 to 63.5) among those who did not receive prior season (2014/15) vaccine. We could not compute VE of current influenza vaccination among those who received prior season (2014/15) due to small sample size.

When modelling VE by time since vaccination among those aged 15 years and older, VE against influenza B ranged from 2.3% at 218 days to 36.6% at 60 days (Figure 3).

Of the 2901 influenza B cases (all ages), 2132 (73.5%) had known B/lineage. Among these, 2.7% were B/Yamagata lineage (57) and 97.3% were B/Victoria lineage (2075). Among the 8 of 9 pilot laboratory study sites that sequenced B‐positive specimens, 321 of 2416 were sequenced (13.3%) (Table 3). Twelve (3.7%) belonged to the genetic group represented by B/Phuket/3073/2013 (Yamagata lineage) group 3. Among the 309 (96.3%) that belonged to the genetic group represented by B/Brisbane/60/2008 (Victoria lineage), all belonged to genetic group 1A, and 308 of them had N129D amino acid substitutions, and one had K56N and V124A amino acid substitutions.

## 4 DISCUSSION

The 2015/16 influenza VE against medically attended ILI due to influenza A(H1N1)pmd09 in the I‐MOVE/I‐MOVE+ multicentre case‐control study in Europe ranged from 13.2% to 55.5% in the total and target population, depending on age group. There was a very low VE or no protective effect against influenza B among the 0‐ to 14‐year‐olds and VE among the 15‐ to 64‐year‐olds among the total and target population ranged from 27.3% to 38.4%.

In the 2015/16 season, twelve study sites contributed to the I‐MOVE multicentre case‐control study and 11 430 individuals were included. This is the largest sample size since the network began in 2008/09. The number of vaccinated patients remains low, even among the target group for vaccination, with 29%‐30% of controls vaccinated. Despite the large sample size, this results in a reduced precision, which is one of the limitations of the study.

Vaccine effectiveness (VE) point estimates against influenza A(H1N1)pdm09 were lower in 2015/16 than in 2014/15, overall and by each age group (54.2, 73.1, 59.7 and 22.4 for all ages, 0‐ to 14‐year‐olds, 15‐ to 59‐year‐olds and those aged 60 and older, respectively). Vaccine effectiveness (VE) point estimates against A(H1N1)pdm09 were also lower in Canada and in the USA, compared to 2013/14, the last year where influenza A(H1N1)pdm09 was a dominant or codominant circulating strain in these countries.^12^, ^13^, ^14^, ^15^ We observed a low influenza A(H1N1)pdm09 VE point estimate among those aged 65 years and older that was not seen in other studies in 2015/16.^14^, ^16^, ^17^ However, in our study, the number of individuals in this age group was low and VE was only adjusted by age and study site.

The results suggest a decrease in VE with time since vaccination against influenza A(H1N1)pdm09 across this long and late season. While the decrease is mild and precision around the estimate is low, this is the first season where we observed this decrease in influenza A(H1N1)pdm09 VE.^11^ A decline in VE against influenza A(H1N1)pdm09 with time across the season was also suggested in the 2015/16 season in Canada.^12^ However, more research on the effects of immunity along the season and the in‐season decline in VE would be useful to validate the results.

In the 2014/15 season, the influenza A(H1N1)pdm09 genetic group 6B dominated, and in 2015/16, a major genetic variant, 6B.1, defined by the HA1 amino acid substitutions S84N, S162N and I216T emerged. For the sites participating in the pilot laboratory project, 90.2% of all sequenced influenza A(H1N1)pdm09 specimens belonged to the 6B.1 genetic group. Antigenic characterisation by haemagglutinin inhibition (HI) assay of circulating influenza A(H1N1)pdm09 viruses from EU/EEA countries using ferret sera indicated that they were antigenically similar to the vaccine virus.^18^ However, 6B.1 viruses were poorly inhibited by some post‐vaccination human serum pools and WHO recommends an influenza A/Michigan/45/2015 (H1N1)pdm09‐like virus (6B.1 genetic group) for the 2017 Southern Hemisphere influenza vaccine.^19^ It is possible that the lower VE point estimate against influenza A(H1N1)pdm09 in the I‐MOVE/I‐MOVE+ study in 2015‐16 may be linked to the changes in the circulating strain compared to the vaccine strain.

The VE point estimate against influenza A(H1N1)pdm09 for those receiving 2015/16 season vaccine only was higher than that among those receiving both 2014/15 and 2015/16 vaccines. While the two estimates are never statistically different from each other, the pattern looks like those from the negative interference hypothesis: that interference from previous season vaccine may be present when consecutive season vaccine components are similar and there is a large antigenic distance between the circulating and vaccine strain.^20^ The 2015/16 and 2014/15 influenza A(H1N1)pdm09 vaccine strains were identical; however, more evidence is needed to determine the antigenic distance between the vaccine strain (A/California/7/2009 (H1N1)pdm09‐like virus) and the 6B.1 circulating genetic group. This pattern was not seen in the 2014/15 season, where vaccine strains were identical and the circulating strain was the 6B genetic group (current and prior season VE point estimates: 47.2% and 52.7%, respectively).^21^ Alternative and also likely explanations for the 2015/16 results may be random variation due to a low vaccination coverage and confounding due to different participant profiles of repeat and single‐season vaccinees.

The VE point estimate of subunit vaccine against influenza A(H1N1)pdm09 was higher than that of split virion among 0‐ to 14‐year‐olds, but the same among 15‐ to 59‐year‐olds. However, precision is low due to small numbers of vaccinated. Age‐specific VE estimates for vaccine groups are not available in previous I‐MOVE study publications and would be useful going forward, numbers of vaccinated allowing.

This is the first season in which the I‐MOVE study could provide representative VE estimates against an influenza genetic group. This represents great progress, although precision around the age‐stratified estimates is low. In the 2015/16 season, there was only one major genetic group circulating. In seasons where two or more genetic clades are cocirculating, more sequencing is needed to obtain a reasonable precision. Precise genetic group‐specific estimates provide important information for interpreting overall VE results and VE results by time since vaccination.

The VE against influenza B was very low or may have conferred no protection among children and was low to moderate among 15‐ to 64‐year‐olds. The differences in VE between age groups were large (*P* < .001). In the context of this effect modification and differential age distributions between studies, due to different healthcare‐seeking behaviours and practitioners included in the study (France, Italy, Germany and Spain include paediatricians in the study), providing a VE among all ages was not appropriate. The age‐specific effect modification and differential age distribution may explain in part why the heterogeneity of study site‐specific estimates among all ages was moderate to high (*I* ^2^ = 55.9%).

In the UK and the USA, the 2015/16 VE against influenza B among children was higher than in the I‐MOVE/I‐MOVE+ multicentre case‐control study. The VE was 56.3% in the UK among those children receiving the (predominantly trivalent) inactivated injectable vaccine, and in the USA, the VE was 64% against B/Yamagata and 56% against B/Victoria among those children receiving the (predominantly quadrivalent) inactivated injectable vaccine.^14^, ^17^ A low VE among children was seen in Finland receiving the (predominantly trivalent) inactivated injectable vaccine (**−**1%).^22^ In the USA, there is a universal vaccination recommendation, and in the UK and Finland, vaccine is recommended in certain age groups in children. However, in the countries participating in the I‐MOVE/I‐MOVE+ multicentre case‐control study, vaccine is recommended only to children with chronic conditions, with the exception of Poland where vaccination is recommended among those aged 6 months to 18 years.^23^, ^24^, ^25^

The low VE against influenza B in children in the I‐MOVE/I‐MOVE+ multicentre case‐control study in the 2015/16 season is in contrast to 2014/15 where VE against influenza B was 62.1% (95% CI: 14.9‐83.1).^21^ While a selection bias among children could explain the low VE against influenza B, the higher VE against influenza A(H1N1)pdm09 among children (31.9%) and the high VE in the 2014/15 season suggest otherwise. Few children in the 2015/16 study were vaccinated with the quadrivalent vaccine (4.4% among those vaccinated children with known vaccine product).

The crude VE against influenza B in those aged 65 years and older was low as observed in the UK (**−**20.2%), in Danish interim estimates (4.1%; hospital‐based patients included) and in the USA (**−**34%; B Flannery, personal communication, 8 March 2017) 2015‐16 season.^16^, ^17^

In the 2015/16 season, the circulating strains were antigenically distinct from the strain selected for the influenza B component in the trivalent influenza vaccine. Nevertheless, there was VE of 27.3% among the 15‐ to 64‐year‐olds. Varying levels of cross‐protection have been reported previously.^26^, ^27^, ^28^ In the 2015/16 season, our VE point estimates are less than 10% among those aged 0‐14 years and those aged 65 years and above. Among older adults and children, the differences observed in VE in a season of mismatch between the vaccine and circulating strains may be explained by a combination of immune system properties specific to children and the elderly, as well as by the role of previous vaccinations and previous infections.

The VE point estimate was higher for subunit vaccine than for split virion vaccine among children, but precision was low. Both estimates were low, indicating that the low VE was not due to a vaccine type‐specific issue. Among 15‐ to 64‐year‐olds, split virion VE point estimate was higher than subunit vaccine, but again precision was low.

In our study, there is residual protection of the prior (2014/15) season vaccine against influenza B among the 15‐ to 64‐year‐olds. The 2014/15 trivalent vaccine also contained a B/Yamagata virus, mismatched with regard to the lineage circulating in 2015/16. Vaccination in current and previous season resulted in a similar VE against influenza B among 15‐ to 59‐year‐olds as vaccination with current vaccine only.

In the 2015/16 influenza season, the results of I‐MOVE/I‐MOVE+ study suggest a lower VE against influenza A(H1N1)pdm09 and influenza B than in previous seasons. Both the low VE against influenza B in children and older adults and the low to moderate VE against influenza B among younger adults may be important in the context of cost‐effectiveness studies looking into recommendations for quadrivalent vaccines and for more precise data need to be collected. Lower VE against influenza A(H1N1)pdm09 in the 2015/16 season, as well as the indications of the effects of previous vaccination seen here and elsewhere need to be evaluated in subsequent seasons together with virological and immunological results.

## CONFLICT OF INTEREST

None.

## APPENDIX 1

### FRANCE

Alessandra Falchi, EA7310, Laboratoire de Virologie, Université de Corse‐Inserm, Corte, France.

Cécile Souty, Thierry Blanchon, Sorbonne Universités, UPMC Univ Paris 06, INSERM, Institut Pierre Louis d’épidémiologie et de Santé Publique (IPLESP UMRS 1136), Paris, France.

### GERMANY

Silke Buda, Udo Buchholz, Ute Preuss, Kerstin Prahm, Department for Infectious Disease Epidemiology, Robert Koch Institute, Berlin, Germany.

Marianne Wedde, Barbara Biere, Alla Heider, Maria Martin, National Reference Centre for Influenza, Robert Koch Institute, Berlin, Germany.

### ITALY

Valeria Alfonsi, Istituto Superiore di Sanità, Rome, Italy.

Maria Rita Castrucci, Simona Puzelli, Annapina Palmieri, National Infuenza Center Istituto, Superiore di Sanità, Rome, Italy.

Elena Pariani, Department of Biomedical Sciences for Health, University of Milan, Italy; Danilo Cereda, Regional Health Authority, Lombardy Region, Italy;

Cinzia Germinario, Maria Chironna, Department of Biomedical Science and Human Oncology, Aldo Moro University of Bari, Italy;

Donatella Tiberti, Regional Reference Service for Infectious Diseases Epidemiology, ASL AL, Alessandria, Italy and Valeria Ghisetti, Department of Infectious Diseases, Amedeo di Savoia Hospital, Turin, Italy;

Maria Grazia Pascucci, Regional Health Authority, Emilia‐Romagna Region, Italy; Paola Affanni, Department of Medicine and Surgery, University of Parma, Italy;

Barbara Camilloni, Department of Experimental Medicine, University of Perugia, Italy;

Pierlanfranco D'Agaro, Department of Medicine, Surgery and Public Health, University of Trieste, Italy; Tolinda Gallo, Regional Health Authority, Friuli Venezia Giulia Region, Italy.

### SPAIN

Fernando Gonzalez Carril, Departamento de Salud, Gobierno del País Vasco, Spain; Rosa Sancho, Subdirección de Salud Pública (Gipuzkoa), Inma Aspiritxaga Gamarra (Vizcaya), Larraitz Etxebarriarteun Aranzabal (Álava) País Vasco, Spain.

Gustavo Cilla, Hospital Universitario Donostia, CIBER Enfermedades Respiratorias (CIBERES), Instituto de Salud Carlos III, Spain.

Manuel Garcia‐Cenoz, Jesus Castilla, Jorge Díaz‐González, Itziar Casado and Iván Martínez‐Baz, Instituto de Salud Pública, Navarra Institute for Health Research (IdiSNA), CIBER Epidemiología y Salud Pública (CIBERESP), Spain.

Ana Navascués and Carmen Ezpeleta, Complejo Hospitalario de Navarra, Spain.

Carmen Quiñones, Eva Martínez, Dirección General de Salud Pública y Consumo de La Rioja, Spain.

Miriam Blasco, Hospital San Pedro de Logroño, Spain.

Jaume Giménez, Juana Vanrell, Servicio de Epidemiología, Dirección General de Salut Pública, Mallorca, Baleares, Spain.

Jordi Reina, Hospital Son Espases de Palma de Mallorca, Spain.

Daniel Castrillejo, Servicio de Epidemiología. DGSC, Consejería de Bienestar Social y Sanidad, Ciudad Autónoma de Melilla, Spain.

Inmaculada Casas, National Centre for Microbiology, National Influenza Reference Laboratory, WHO‐National Influenza Centre, Instituto de Salud Carlos III, Majadahonda, Spain.

Concha Delgado, Jesús Oliva, Centro Nacional de Epidemiología. CIBERESP, Instituto de Salud Carlos III, Madrid, Spain.

### HUNGARY

Zita Vizler National Centre for Epidemiology, Hungary.

Éva Hercegh, Bálint Szalai, National Centre for Epidemiology, Influenza Virus Laboratory Hungary.

### SWEDEN

Åsa Wiman, The Public Health Agency of Sweden, Unit for Laboratory Surveillance of Viral Pathogens and Vaccine Preventable Diseases, Sweden.

### IRELAND

Anita Kelly, HSE‐Health Protection Surveillance Centre, Ireland.

Michael Joyce, Claire Collins, Irish College of General Practitioners, Ireland.

Cillian de Gascun, Jeff Connell, Grainne Tuite, Margaret Duffy, Joanne Moran, Linda Dunford, National Virus Reference Laboratory, University College Dublin, Ireland.

### POLAND

Lidia Brydak, National Institute of Public Health, National Institute of Health, Warsaw, Poland.

### THE NETHERLANDS

Frederika Dijkstra, Wim van der Hoek, Marit de Lange, Adam Meijer, Pieter Overduin, Anne Teirlinck, National Institute for Public Health and the Environment (RIVM), Bilthoven, the Netherlands.

Gé Donker, Netherlands Institute for Health Services Research (NIVEL), Utrecht, the Netherlands.

### PORTUGAL

Verónica Gomez, Ana Paula Rodrigues, Baltazar Nunes, Departamento de Epidemiologia, Instituto Nacional de Saúde Dr. Ricardo Jorge, Lisbon, Portugal.

Pedro Pechirra, Paula Cristóvão, Patrícia Conde, Inês Costa, Departamento de Doenças Infeciosas, Instituto Nacional de Saúde Dr. Ricardo Jorge, Lisbon, Portugal.

### ROMANIA

Daniela Pitigoi, Emilia Lupulescu, Carmen Maria Cherciu, Cantacuzino Institute, National Institute of Research – Development for Microbiology and Immunology, Bucharest, Romania.

### CROATIA

Bernard Kaić, Iva Pem Novosel, Goranka Petrović, Croatian Institute of Public Health, Zagreb, Croatia.
